# Elevated monocyte-to-lymphocyte ratio, C-reactive protein and further inflammatory parameters as potential biomarkers of suicide risk in bipolar I disorder

**DOI:** 10.3389/fpsyt.2025.1648202

**Published:** 2025-12-08

**Authors:** Borbála Pethő, Róbert Herold, Diána Simon, Márton Áron Kovács, Tünde Tóth, Noémi Albert, Dóra Hebling, András Sándor Hajnal, Tímea Csulak, Márton Herold, Tamás Tényi

**Affiliations:** 1 Department of Psychiatry and Psychotherapy, Clinical Center, Medical School, University of Pécs, Pécs, Hungary; 2 Department of Immunology and Biotechnology, Clinical Center, Medical School, University of Pécs, Pécs, Hungary; 3 Department of Anatomy, Medical School, University of Pécs, Pécs, Hungary

**Keywords:** biomarker, inflammation, laboratory parameters, bipolar I disorder, monocyte-to-lymphocyte ratio, suicidality

## Abstract

Suicide is an unresolved issue in psychiatry to this day. Suicide risk (SR) is particularly high for psychiatric patients with bipolar I disorder (BD). Recent studies suggest an immunological dysregulation in the background. In our retrospective study, we investigated laboratory parameters of BD in-patients (*n* = 116) between January 2020 and June 2024. Data was collected regarding the following parameters: white blood cell, neutrophil, lymphocyte, monocyte and platelet count, monocyte-to-lymphocyte (MLR), neutrophil-to-lymphocyte (NLR) and platelet-to-lymphocyte ratio (PLR), C-reactive protein (CRP), erythrocyte sedimentation rate (ESR), red blood cell distribution width (RDW) and mean platelet volume (MPV). Individuals with recent (≤ 48 hours prior) suicide attempt (SA) (*n* = 21) and with past (> 48 hours prior) SA (*n* = 16) represented the high SR group (*n* = 37). BD patients with no history of SA composed the intermediate SR group (*n* = 79). We found a significant increase in MLR, monocyte count, CRP and ESR in patients with recent SA compared to those with no history of SA. Comparing high and intermediate SR patients, MLR, monocyte count, CRP and ESR remained elevated in the former group. As implied by previous research, immunological mechanisms may contribute to the emergence of suicidality. Investigating BD patients as the subgroup at significant risk, changes in certain inflammatory markers further strengthen the assumption of immunological processes in the background of suicidality, and these parameters may serve as potential future biomarkers of SR.

## 1 Introduction

Although the global health burden of suicide is severe, as it takes more than 700.000 lives every year (1), determining the risk in the clinical setting remains a challenge to this day. The emergence of suicidality (2) – with the term including suicidal ideations, plans and attempts – is a complex mechanism, with several contributing factors. Proximal or acute risk factors include substance abuse, interpersonal events and negative affective states, such as anxiety, agitation or insomnia. Distal risk factors or predisposing vulnerabilities may be genetic characteristics, childhood traumatic events or a past suicide attempt (3). Among the latter, the presence of a psychiatric illness contributes greatly to the emergence of suicidal behavior (4) – according to a research conducted in 2025, excess life-years lost as a result of mental disorders range from 5.4 to 14.8 years (5). Patients with bipolar I disorder (BD) are at significant risk (6): compared to healthy individuals, psychiatric patients with mood disorders have an 8.1-fold chance of dying by suicide (7). Although once suicidal ideations emerge, 60% of attempts are made in the following year (8), individuals often fail to disclose suicidal plans when meeting with healthcare professionals (9). The current risk stratification methods in use are mainly subjective and self-report based (10), and their accuracy may be diminished by the lack of trust or cooperation from patients in a state of suicide crisis. Consequently, there is a great need for an objective supplement to the existing methods. According to novel research findings, certain laboratory parameters may be reliable indicators of suicide risk (SR) (11).

Our research group has previously found monocyte-to-lymphocyte ratio (MLR) and other inflammatory markers to be potential biomarkers of SR in patients with major depressive disorder (MDD) (12). Considering that 25-60% of BD patients attempt suicide at least once in their lifetime, individuals living with BD are one of the most vulnerable population regarding suicidality (13).

Determining SR remains a challenge for clinicians in psychiatric care. Alterations in the number and ratio of inflammatory cells have been proposed as potential biomarkers of SR, therefore our aim was to investigate the efficiency of these parameters in determining acute and long-term SR among patients with BD. We hypothesized that comparing patients with recent suicide attempt (SA) to those with no history of SA, and individuals with high SR to those with intermediate SR, differences in the number of immunological parameters would signal the vulnerability of each group.

## 2 Materials and methods

Data was collected retrospectively from psychiatric in-patients (*n* = 116) diagnosed with bipolar I disorder according to the Diagnostic and Statistical Manual of Mental Disorders, Fifth Edition (DSM-5). The investigation was performed at the Department of Psychiatry and Psychotherapy, Medical School, Clinical Center, University of Pécs between January 2020 and June 2024. We collected the number of neutrophil granulocytes, monocytes, lymphocytes, platelets and white blood cells (WBC). Furthermore, we included neutrophil-to-lymphocyte ratio (NLR), MLR, platelet-to-lymphocyte ratio (PLR), C-reactive protein (CRP), erythrocyte sedimentation rate (ESR), red blood cell distribution width (RDW) and mean platelet volume (MPV). We divided participants into three groups based on the presence of a previous SA: recent (≤ 48 hours prior) (*n* = 21), past (> 48 hours prior) attempters (*n* = 16) and individuals with no history of SA (*n* = 79). Further grouping patients according to SR, participants with recent or past SA represented the high SR group (*n* = 37) and BD patients with no history of SA were considered as individuals with intermediate SR (*n* = 79). All recent attempts were carried out by self-poisoning with benzodiazepines. All participants were of Caucasian ethnicity, with age ranging from 22 to 77 years old.

The exclusion criteria included acute or chronic inflammatory illnesses, autoimmune diseases, hematological or oncological conditions and current treatment with anti-inflammatory or immunosuppressive medication. Due to the confounding effects of trauma-related inflammation, individuals with an attempt by causing physical injury were excluded from the recent SA group. There were no significant differences regarding age and gender between the patient groups. Although previous studies have found a correlation between chronic diseases and inflammatory markers (14, 15), and the presence of these conditions may have had an effect on the investigated parameters, we did not observe any significant differences regarding laboratory parameters between patients with a positive and negative anamnesis of a concomitant medical condition (hypertension, diabetes mellitus).

All of the participants had been undergoing pharmacological treatment for at least 1 month. Subsets of them received AP, AD therapy and mood stabilizers. AP treatment included first, second and third generation agents. AD treatment consisted of selective serotonin reuptake inhibitor (SSRI), serotonin-noradrenaline reuptake inhibitor (SNRI), noradrenaline and specific serotonergic antidepressant (NaSSA), serotonin antagonist and reuptake inhibitor (SARI), selective serotonin reuptake enhancer (SSRE), multimodal and tricyclic AD medication. Mood stabilizers incorporated lithium, carbamazepine, valproate or lamotrigine. Sociodemographic and clinical characteristics of the patient groups are summarized in **Table 1**.

**Table 1:** Sociodemographic and clinical characteristics of the patient groups.

Variable	Total sample (*n* = 116)	Recent SA (*n* = 21)	Past SA (*n* = 16)	No history of SA (*n* = 79)	High SR (*n* = 37)	Intermediate SR (*n* = 79)
Males (*n* [%])	43 (37, 07)	4 (19, 05)	2 (12, 50)	37 (46, 84)	6 (16, 22)	2 (12, 50)
Females (*n* [%])	73 (62, 93)	17 (80, 95)	14 (87, 50)	42 (53, 16)	31 (83, 78)	14 (87, 50)
Age (mean ± SD)	49, 78 ± 16, 23	57, 19 ± 10, 67	57, 75 ± 11, 95	46, 19 ± 17, 05	57, 43 ± 11, 08	46, 19 ± 17, 05
Concomitant diabetes mellitus (*n* [%])	18 (15, 55)	6 (28, 57)	5 (31, 25)	7 (8, 86)	11 (29, 73)	5 (31, 25)
Concomitant hypertension (*n* [%])	41 (35, 34)	11 (52, 38)	7 (43, 75)	23 (29, 11)	18 (48, 65)	7 (43, 75)
Depressive episode on admission (*n* [%])	68 (58, 62)	21 (100, 00)	8 (50, 00)	39 (49, 37)	29 (78, 38)	39 (49, 37)
Manic episode on admission (*n* [%])	33 (28, 45)	0 (0, 00)	5 (31, 25)	28 (35, 44)	5 (13, 51)	28 (35, 44)
Mixed episode on admission (*n* [%])	15 (12, 93)	0 (0, 00)	3 (18, 75)	12 (15, 19)	3 (8, 11)	12 (15, 19)
AP treatment (*n* [%])	70 (60, 34)	13 (61, 90)	11 (68, 75)	46 (58, 23)	24 (64, 86)	46 (58, 23)
AD treatment (*n* [%])	47 (40, 52)	12 (57, 14)	6 (37, 50)	29 (36, 71)	18 (48, 65)	29 (36, 71)
MS treatment (*n* [%])	59 (50, 86)	13 (61, 90)	10 (62, 50)	36 (45, 57)	23 (62, 16)	36 (45, 57)

AP, antipsychotic; AD, antidepressant; SA, suicide attempt; SR, suicide risk; SD, standard deviation; MS, mood stabilizer.

Blood draw was carried out on the morning after admission. The results were evaluated via routine laboratory assays at the Department of Laboratory Medicine, Medical School, Clinical Center, University of Pécs. For the statistical analysis, GraphPad Prism version 10.0 Windows program (GraphPad Software, San Diego, CA, USA, www.graphpad.com, accessed in 2024) was used. Statistical operations were carried out first by performing a descriptive analysis and then by determining the distribution of samples via a variety of tests (Shapiro-Wilk, Kolmogorow-Smirnow, Anderson-Darling, D’Agostino-Pearson) and mainly by considering the Q-Q plot. Outliers were identified using the robust regression and outlier removal (ROUT) method and excluded from further statistical analyses. For comparisons between groups, Mann-Whitney U test was used. In all cases, *p* ≤ 0.05 was considered statistically significant.

## 3 Results

### 3.1 Inflammatory parameters in BD patients with recent SA and no history of SA

**Figure 1:**
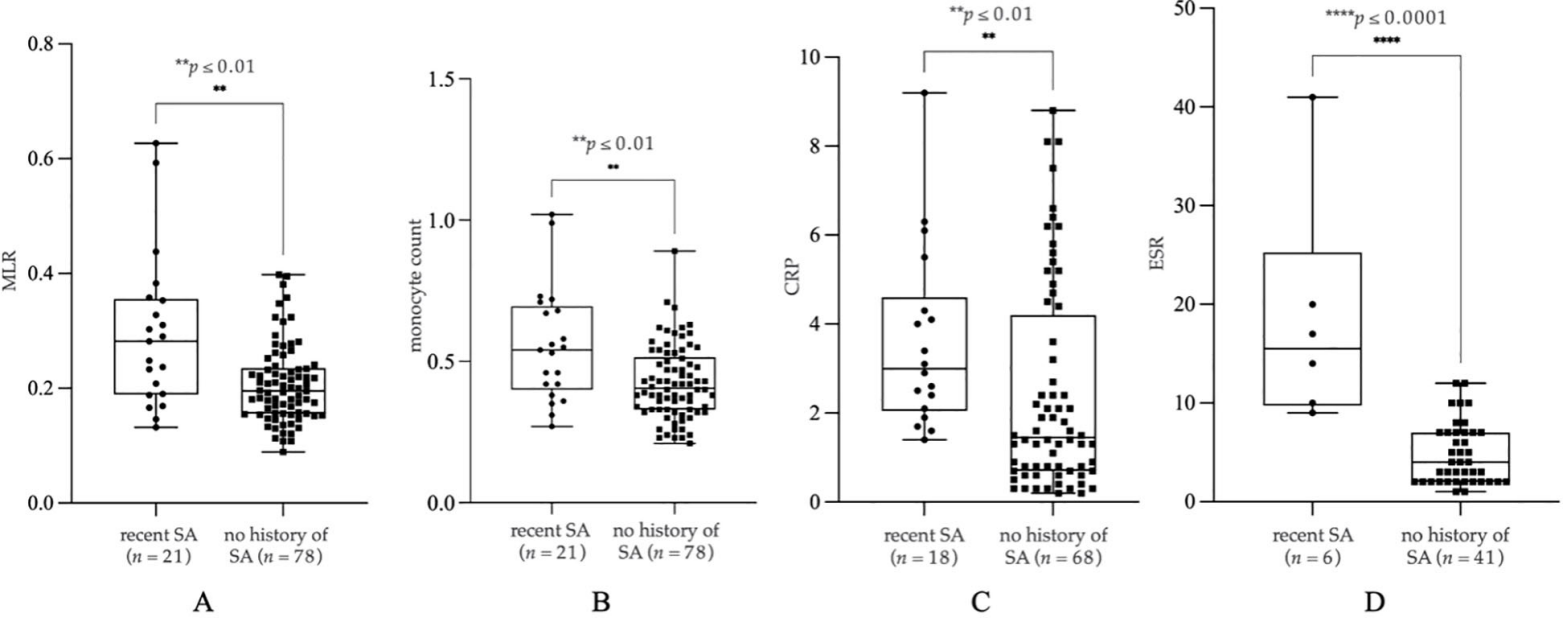
Monocyte count **(A)**, monocyte-to-lymphocyte ratio (MLR) **(B)**, C-reactive protein (CRP) **(C)** and erythrocyte sedimentation rate (ESR) **(D)** in patients with no history of suicide attempt (SA) and recent SA. The box plot diagram represents the interquartile range and median values. Whiskers indicate the most extreme observations. The individual values are presented with black dots (patients with recent SA, *n*_MLR_ = 21, *n*_monocyte_ = 21, *n*_CRP_ = 18, *n*_ESR_ = 6) and squares (no history of SA, *n*_MLR_ = 78, *n*_monocyte_ = 78, *n*_CRP_ = 68, *n*_ESR_ = 41). For statistical analysis, the Mann-Whitney U test was used. **p ≤ 0.01, ****p ≤ 0.0001.

We found a significant increase in MLR (*p* = 0.0021, *n* = 21, mean: 0.29 ± 0.13, median: 0, 28, **Figure 1A**), monocyte count (*p* ≤ 0.0045, *n* = 21, mean: 0.56 ± 0.20 G/l, median: 0, 54, **Figure 1B**), CRP (*p* = 0.0036, *n* = 18, mean: 3.62 ± 2.05 mg/l, median: 3, 00, **Figure 1C**) and ESR (*p* ≤ 0.0001, *n* = 6, mean: 18.50 ± 11.78 mm/h, median: 15, 50, **Figure 1D**) in patients with recent SA compared to individuals with no history of SA (*n*_MLR_ = 78, mean_MLR_: 0.21 ± 0.07, median_MLR_: 0, 19; *n*_monocyte_ = 78, mean_monocyte_: 0.42 ± 0.13 G/l, median_monocyte_: 0, 41; *n*_CRP_ = 68, mean_CRP_: 2.44 ± 2.33 mg/l, median_CRP_: 1, 45; *n*_ESR_ = 41, mean_ESR_: 4.76 ± 3.09 mm/h, median_ESR_: 4, 00). We have included further statistical values in **Table 2**.

**Table 2:** Laboratory parameters showing significant differences between the patient groups.

Patient groups	Diagnostic value	MLR	Monocyte count	CRP	ESR
Recent SA vs. no history of SA	AUC	0, 72	0, 699	0, 72	0, 97
	CI	0, 58-0, 85	0, 57-0, 83	0, 61-0, 83	0, 91-1, 00
	Significance (*p*)	0, 0024	0, 005	0, 0042	0, 0003
High SR vs. intermediate SR	AUC	0, 64	0, 63	0, 62	0, 79
	CI	0, 53-0, 76	0, 51-0, 74	0, 51-0, 73	0, 63-0, 96
	Significance (*p*)	0, 0124	0, 0298	0, 05	0, 0015

AUC, area under curve; CI, confidence interval; CRP, C-reactive protein; ESR, erythrocyte sedimentation rate; MLR, monocyte-to-lymphocyte ratio; SA, suicide attempt; SR, suicide risk.

There were no significant differences between the two groups regarding neutrophil granulocyte, lymphocyte and platelet count, WBC, NLR, PLR, RDW and MPV.

### 3.2 Inflammatory parameters in BD patients with high SR and intermediate SR

**Figure 2:**
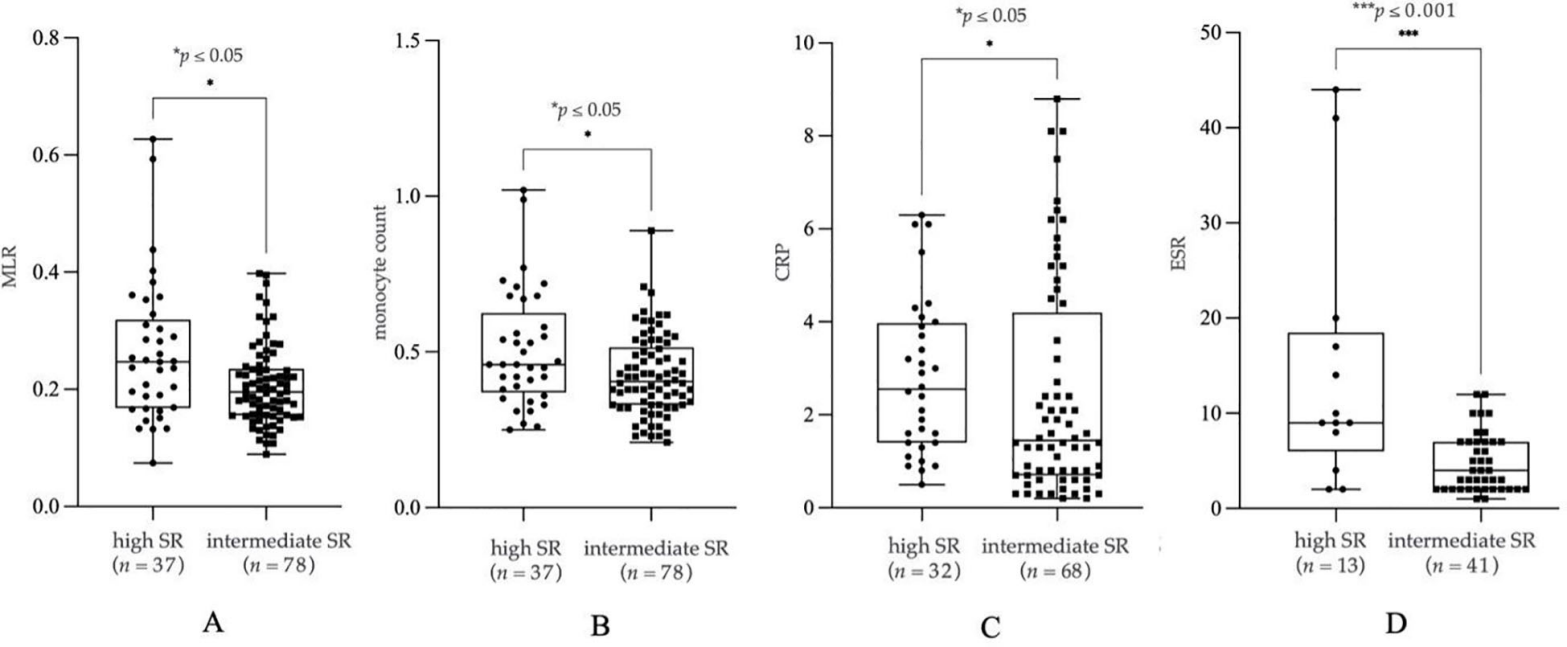
Monocyte count **(A)**, monocyte-to-lymphocyte ratio (MLR) **(B)**, C-reactive protein (CRP) **(C)** and erythrocyte sedimentation rate (ESR) **(D)** in patients with high suicide risk (SR) and intermediate SR. The box plot diagram represents the interquartile range and median values. Whiskers indicate the most extreme observations. The individual values are presented with black dots (high SR, *n*_MLR_ = 37, *n*_monocyte_ = 37, *n*_CRP_ = 32, *n*_ESR_ = 13) and squares (intermediate SR, *n*_MLR_ = 78, *n*_monocyte_ = 78, *n*_CRP_ = 68, *n*_ESR_ = 41). For statistical analysis, the Mann-Whitney U test was used. *p ≤ 0.05, ***p ≤ 0.001.

We found a significant increase in MLR (*p* = 0.0120, *n* = 37, mean: 0.26 ± 0.12, median: 0, 25, **Figure 2A**), monocyte count (*p* = 0.0293, *n* = 37, mean: 0.51 ± 0.19 G/l, median: 0, 46, **Figure 2B**), CRP (*p* = 0.049, *n* = 32, mean: 2.80 ± 1.67 mg/l, median: 2, 55, **Figure 2C**) and ESR (*p* = 0.0009, *n* = 13, mean: 14.54 ± 13.49 mm/h, median: 9, 00, **Figure 2D**) in patients with high SR compared to participants with intermediate SR (*n*_MLR_ = 78, mean_MLR_: 0.21 ± 0.07, median_MLR_: 0, 19; *n*_monocyte_ = 78, mean_monocyte_: 0.42 ± 0.13 G/l, median_monocyte_: 0, 41; *n*_CRP_ = 68, mean_CRP_: 2.44 ± 2.33 mg/l, median_CRP_: 1, 45; *n*_ESR_ = 41, mean_ESR_: 4.76 ± 3.09 mm/h, median_ESR_: 4, 00). We have summarized further statistical values in **Table 2**.

We found no significant differences between the two groups regarding neutrophil granulocyte, lymphocyte and platelet count, WBC, NLR, PLR, RDW and MPV.

### 3.3 Effects of pharmacological treatment

Previous research has suggested anti-inflammatory effects of AP (16) and AD (17) pharmacotherapy, therefore we investigated changes of the parameters related to these types of medication. Comparing treated and untreated patients, we found no significant differences regarding AP treatment (*p* > 0.05). We found no significant differences between individuals receiving AD pharmacotherapy and untreated participants (*p* > 0.05).

## 4 Discussion

Investigating BD patients, we found monocyte count, MLR, CRP and ESR to be associated with elevated SR, suggesting inflammatory processes in the background of suicidality. According to the AUC values, the majority of these parameters have an acceptable diagnostic performance and ESR has an outstanding diagnostic accuracy in differentiating patients with a recent SA.

An increasing number of novel investigations have proposed that an immunological dysregulation may contribute to the emergence of suicidal behavior (18–20). The exact pathomechanism remains unclear, however, studies have shown alterations in the levels of inflammatory markers in association with suicidality: increased levels of CRP (21) and proinflammatory cytokines (22) were found in patients with suicidal ideations or attempts. These changes were not limited to peripheral blood: decreased concentration of anti-inflammatory cytokines has been found in the cerebrospinal fluid of suicidal patients compared to healthy controls (23), and a postmortem study on brain tissue revealed increased proinflammatory cytokine levels and decreased anti-inflammatory cytokine concentration in suicide completers compared to healthy individuals (24).

There is a possible link between affective disorders and altered immunological processes, and it has been suggested that an elevated level of inflammatory response in the background of MDD (25) or BD (26, 27) may lead to the emergence of suicidality in patients with these conditions. Considering the phenomenon of sickness behavior, which is a physiological adaptive response to infection, many similarities may be found between the symptomatology of a depressive episode and that of an inflammatory state, such as deprived mood, decreased activity, loss of appetite and changes in sleep (28). These symptoms have been observed in animal studies (29) and in patients receiving immune therapy (30), and the inflammatory response is mediated by cytokines (31), altering the functioning of the hypothalamus-pituitary-adrenal axis (32), causing affective and behavioral changes (33, 34) and deficits in cognitive processes (35).

Immunological mechanisms are carried out and therefore may be measured by the alterations in the levels of not only cytokines, but certain blood cells. Changes of monocyte count and MLR have been associated with both MDD (36) and BD (37–39). Furthermore, MLR has been proved to be elevated in suicidal MDD patients compared to non-suicidal individuals (40), inferring to a different level of immunological processes related to suicidality in patients with mood disorders. Elevated levels of CRP have also been found in patients with mood disorders having attempted suicide compared to non-suicidal individuals (41). In a previous study, ESR has been associated with suicidal ideations in patients with MDD (42). Our results are in accord with these findings: we have detected a significant elevation regarding monocyte count, MLR, CRP and ESR in BD patients with recent SA compared to those with no history of SA. As we compared all high SR patients – participants with a recent or a past attempt – to intermediate SR individuals, all of these significant differences persisted. Therefore, these parameters are not only associated with acute SR, but they may be potential indicators of long term suicidal vulnerability. In order to assess the reliability of these markers in the clinical practice, further investigation is required, however, as part of a routine laboratory examination, these parameters hold the promise of becoming an accessible, reproducible, cost-effective, objective supplement to the methods currently in use, possibly leading to more precise evaluation of patients at risk for suicide in the future.

## 5 Conclusion

We have found monocyte count, MLR, CRP and ESR to be possible indicators of acute and long-term SR in patients with BD. To the best of our knowledge, this is the first research to find elevated monocyte count and MLR in association with SR in BD patients. In order to specify the exact immunological mechanisms and potential anti-inflammatory effects resulting in the alterations of these parameters, and to evaluate the effectivity of these markers in the clinical practice, more in-depth research is needed.

## 6 Limitations

Our research has not entailed patients with bipolar II disorder, although determining the risk of suicide for this subgroup would be of key importance for further investigation. Cytokines have not been included in this research, as we planned to investigate cost-effective laboratory parameters that could be measured routinely in the clinical setting. We have not enrolled healthy controls, as we intended to focus on the changes related specifically to suicidality, not to the presence of a mental disorder. Furthermore, patients with a psychiatric illness, especially those suffering from affective disorders are a vulnerable subgroup in the general population regarding suicide risk, therefore their assessment requires more precision. However, investigations on the general population may provide further important information. We only examined individuals committing suicide via self-poisoning with benzodiazepines – although this selection limited the confounding effect of inflammation stemming from physical injury, not all patients with SA are represented in our investigation. We have limited information about the participants regarding smoking, which is known to have a possible proinflammatory effect. Data on body mass index (BMI) was not included, although it may modulate inflammatory processes. A further limitation of this study has been the AD and AP medication received by the participants – studies have shown anti-inflammatory effects related to these types of pharmacotherapy, however, when comparing cell numbers and ratios of treated and untreated patients with BD, we did not find any significant differences between the two groups. There were no significant differences between either the recent SA and no history of SA groups or the high and intermediate SR groups regarding neutrophil granulocyte, lymphocyte and platelet count, WBC, NLR, PLR, RDW and MPV – a further limitation of this study may have been the number of participants, which may not have showcased more delicate differences between the patient groups. Sample size calculation was not performed for this investigation.

## Data Availability

The original contributions presented in the study are included in the article/supplementary material. Further inquiries can be directed to the corresponding author.
